# Interpretation of Single-Molecule Force Experiments on Proteins Using Normal Mode Analysis

**DOI:** 10.3390/nano11112795

**Published:** 2021-10-22

**Authors:** Jacob Bauer, Gabriel Žoldák

**Affiliations:** 1 Institute of Molecular Biology, Slovak Academy of Sciences, Dúbravská cesta 21, 845 51 Bratislava, Slovakia; 2 Center for Interdisciplinary Biosciences, P. J. Šafárik University, Technology and Innovation Park, Trieda SNP 1, 041 54 Košice, Slovakia

**Keywords:** single-molecule force spectroscopy, single-molecule optical trap experiments, normal mode analysis, computational chemistry

## Abstract

Single-molecule force spectroscopy experiments allow protein folding and unfolding to be explored using mechanical force. Probably the most informative technique for interpreting the results of these experiments at the structural level makes use of steered molecular dynamics (MD) simulations, which can explicitly model the protein under load. Unfortunately, this technique is computationally expensive for many of the most interesting biological molecules. Here, we find that normal mode analysis (NMA), a significantly cheaper technique from a computational perspective, allows at least some of the insights provided by MD simulation to be gathered. We apply this technique to three non-homologous proteins that were previously studied by force spectroscopy: T4 lysozyme (T4L), Hsp70 and the glucocorticoid receptor domain (GCR). The NMA results for T4L and Hsp70 are compared with steered MD simulations conducted previously, and we find that we can recover the main results. For the GCR, which did not undergo MD simulation, our approach identifies substructures that correlate with experimentally identified unfolding intermediates. Overall, we find that NMA can make a valuable addition to the analysis toolkit for the structural analysis of single-molecule force experiments on proteins.

## 1. Introduction

Studying protein molecules *in singulo* has revealed many details on their folding mechanism, protein–protein interactions and assembly [1,2,3,4,5,6,7,8]. Single-molecule optical trap experiments, a technique for conducting single-molecule force spectroscopy, are a relatively recent development that allows protein folding and unfolding to be explored under the application of mechanical force [9]. By probing individual molecules, they allow rare events and intermediate states to be resolved that might be obscured in ensemble experiments. In optical trap experiments, the protein to be studied is attached to two micron-sized polystyrene beads using double-stranded DNA tethers. One of these beads is usually held fixed while the other is in an optical trap which is moved in order to apply force to the protein. The DNA tethers are attached to the protein through disulfide linkages and allow the force to be applied across the molecule at freely chosen locations. They also allow the protein to be isolated from non-specific interactions.

There are typically three different protocols of experimental probing that can be performed. The first is a force-ramp experiment, in which the two beads are moved apart and together alternately at a constant pulling speed, which increases and decreases the force on the molecule. The resulting experimental outcomes are force–extension curves. These curves are interrupted by sudden “rips” where unfolding and refolding events occur. Careful examination of these rips can identify folding and unfolding intermediates. The Crooks fluctuation theorem can be used to estimate the equilibrium free energies involved in the folding–unfolding transitions [10,11], and the kinetic parameters can also be recovered [12,13]. The second type is a constant-force experiment. There are two different kinds of constant-force experiments. In a force-jump experiment, the protein is subjected to one force and then jumped to a second force where folding or unfolding is expected to occur and folding or unfolding can be followed at each of these steps. In “hopping” experiments, the force is maintained at a single value, and the trap position is monitored to follow the folding and refolding of the protein as it “hops” between different states. The third type of experiment is similar to the second, but the trap’s position is held constant rather than the applied force.

All three experiment types result in a one-dimensional force spectrum that can be used to identify changes in conformation and folding state, including the presence of (un)folding intermediates, but which must be interpreted by other means to extract structural characteristics. To determine the contour length of the unfolded portion of the molecules, a contour-length transformation using a worm-like chain model is frequently employed [14]. This can give an idea of how many residues remain folded or unfolded for a given intermediate, but does not usually allow their identity to be inferred. Probably the best way to interpret the results of these experiments is to use steered (coarse-grained) molecular dynamics (MD) simulation, using, for example, a self-organized polymer model [15,16]. This explicitly models both the protein and the experimental conditions and can be used to directly determine the nature of the new conformations and (un)folding intermediates. However, because of the computational expense of the method, most steered MD simulations use stretching velocities that are substantially higher than those used in the pulling experiments themselves. This means that the forces calculated from the simulations will be higher than those from experiments, raising the question of whether the unfolding events captured by simulation truly corresponded to those measured experimentally [17]. It does seem that faster pulling can alter the nature of the protein’s elastic response [18], but studies have also shown that unfolding pathways are not necessarily sensitive to pulling forces and speeds [19,20]. Recently, high-speed force spectroscopy was developed, which reduces the gap between the experimental and simulated velocities [21], and coarse-grained models were successfully used to examine the mechanical properties of proteins near experimental pulling velocities with reasonable computational time [22]. Nevertheless, for the majority of force spectroscopy experiments, the computational expense of these simulations will still be the limiting step for any study that relies on it.

Faced with this issue, we wondered if it might be possible to acquire some of the insights provided by steered MD simulation more easily and more quickly using normal mode analysis (NMA). NMA is often used to describe the flexible states available to a protein, but only about a minimum-energy, equilibrium conformation [23]. Unlike MD simulation, which is stochastic, NMA produces analytical solutions to the equations of motion: given an initial set of positions and velocities, it allows us to calculate where each atom of a given system will be at any subsequent time subject to the small oscillation approximation, and NMA has been shown to reliably reproduce the motions described by principal component analysis of MD simulations [24,25,26,27].

There are, however, at least two difficulties involved in applying NMA to this situation. First, the results of NMA are strongly dependent on the conformation of the starting structure, and it is obvious that the structure of the protein caught in the optical trap apparatus will not be that found in its crystal structure. The second is that a protein in this experimental arrangement is not in any sort of meaningful energy minimum: there is plainly a net force acting upon it, and even if it might be claimed to exist in some sort of equilibrium, the small oscillation approximation would likely not apply. However, our goal is not to explicitly model what occurs within the experimental apparatus, but rather, to gain some qualitative insight into how the protein might behave under the conditions that occur there. To achieve this, it is enough to answer some related questions about the nature of the protein and its internal structure. For example, what are the structurally weakest parts of the molecule? What are the most flexible, and therefore, the most labile parts? For multi-domain proteins, which modes move the domains apart and might be enhanced as a result of the applied force? As shown below, answers to these questions do allow us to generally come to the same conclusions that were reached by more computationally demanding methods.

Below, we will describe a general procedure for using NMA to interpret the results of single-molecule optical trap experiments. We will apply this procedure to four previously published results to evaluate its uses and limitations. We find that, despite its limitations, NMA does allow a fair amount of insight into the behavior of these molecules in the published experiments to be gathered.

## 2. Materials and Methods

### 2.1. General Considerations

Normal mode analysis is a computational technique, based on the physics used to describe small oscillations, that can be used to describe the flexible states accessible to a protein or other molecule in an energy minimum conformation. In such a conformation, the potential energy of the system can be represented by the second order term in a power series expansion:

$$V\left(q\right)=\frac{1}{2}\left(\frac{\partial^{2}V}{\partial q_{i}\partial q_{j}}\right)\eta_{i}\eta_{j}=\frac{1}{2}\eta_{i}V_{ij}\eta_{j}$$

Here, *q* refers to generalized coordinates, *q_i_* and *q_j_* are the instantaneous positions of components *i* and *j*, *η_i_* is the deviation of component *i* from its minimum energy configuration, and *V_ij_* is the Hessian matrix. *V(q)* is the potential energy equation of the system and can take the form of either one of the commonly used molecular dynamics force fields or an elastic network model (ENM). In an ENM, the protein is represented by a network of beads with each bead corresponding to a Cα atom of the protein, and the pairwise interactions between all Cα atoms within a distance *R_c_* of each other are modeled by a harmonic potential with a uniform force constant *C*:

$$V\left(q\right)=\underset{d_{ij}<R_{c}}{\sum }C{\left(d_{ij}-d_{ij}^{0}\right)}^{2}$$

where *d_ij_* is the distance between components *i* and *j* and *d_ij_*^0^ is the distance in the equilibrium structure. The cutoff distance *R_c_* is normally between 8 and 20 Å.

According to the small oscillation approximation, a system being studied is assumed to fluctuate so little that it behaves, for all practical purposes, as a solid. Proteins have been experimentally observed to behave in this manner, but only at temperatures below 100–200 K [28,29,30]. A natural consequence of this is that a more solid or dense part of the protein is expected to be less flexible and structurally stronger than a less tightly packed part. The more densely packed regions of the protein can be visualized by identifying those residues that form the buried core of a protein. Perhaps the simplest way to do this is to identify all the solvent-exposed surface residues and then take their complement. This gives a similar result to that seen for T4 Lysozyme in the figure referenced in Section 3.1 below. NMA shows that the residues marked as forming part of the core are actually among the least flexible in the protein. This observation seems to be general for the four proteins studied here. A representation such as that shown in panel a of the figure referenced in Section 3.1 below is, by itself, often enough to identify joints between domains and the structurally weaker areas of the molecule. It can also be used qualitatively to describe the likely effect of applying force to the molecule, especially when the force is applied to the N- and C-termini. As for the Hsp70 nucleotide-binding domain and the GCR, it can also often be used to suggest whether NMA should be applied to truncated forms of the molecule. The character of the residues making up the packed core may also be used to qualitatively infer something about the strength of the packing (for example, a core composed mostly of interlocking branched residues is likely to be both more structurally rigid and mechanically stronger than one composed largely of alanine residues).

**Figure 1:**
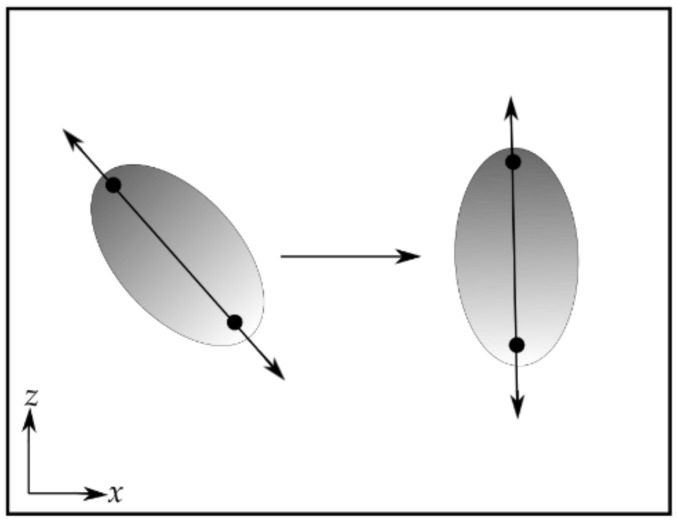
A schematic illustrating the rotation of a protein of interest is aligned with one of the principal coordinate axes. The protein being studied (gray ellipse) is normally in a random orientation. It should be rotated so that the direction of applied force (the lines with double arrowheads passing through the ellipses) is at least roughly parallel to one of the principal coordinate axes (the *z*-axis in this example). The black circles represent the site of attachment of the DNA tethers.

Before NMA, it may also be advantageous, especially for multi-domain proteins, to rotate the molecule so that the direction of applied force is along one of the principal coordinate axes. This will allow the modes that separate domains or other parts of the molecule in the direction of the applied force to be more easily identified (Figure 1).

The types of analyses employed will depend on the nature of the protein being studied and the type of experiment. Generally, the most useful analyses include the square fluctuations for each residue, and the relative mechanical stiffness of each residue [31,32]. Together, these will show which parts of the molecule are the most and the least flexible and may suggest, depending on the nature of the experiment, which parts of the protein are likely to come apart first.

In selecting the individual modes to examine, it is best to pay attention to both their collectivity, how many atoms take part in a given mode, and their rank order (lower-frequency modes occur at the top of the list, higher-frequency modes closer to the bottom). Modes with greater collectivity move greater numbers of atoms, and modes of lower rank order tend to move groups of atoms with greater connectivity. Often, these two factors go together, and the lower-frequency modes move larger numbers of connected atoms so that they describe the rotation and translation of complete domains. It sometimes happens that higher-frequency modes will also have large collectivity, but these modes often reflect the concerted motion of disparate, unconnected parts of the molecule, for example, the concerted motion of several surface loops, but with not domain movements. In all cases, the first 10 non-zero normal modes should be examined, and the 10 normal modes with the highest collectivity of the top 50 should also be checked.

We used both ProDy [33,34] and the off-line components of the ElNémo web server [35] for these experiments. The results were largely the same in both cases.

Our procedure for interpreting the results of single-molecule force spectroscopy proceeds through five steps.

### 2.2. Procedure

1. Examine the structure to identify the expected effects of the force on its general form, taking into consideration the size and location of the packed core. Decide whether or not additional truncated forms should be studied.
2. Rotate the protein so that the direction of applied force in the experiments will be along one of the principal coordinate axes. This will allow those modes that separate the domains or subdomains to be more easily identified. In the case of all four of the proteins described below, it was most convenient to align the protein to the *z*-axis, though any other axis might have been used.
3. Calculate the normal modes. This can be achieved using either conventional all-atom NMA or one of the coarse-grained ENMs. The first 50 non-zero normal modes will be sufficient for most purposes, but all modes will be needed to calculate the mechanical stiffness.
4. Examine the results. For single-domain proteins, plots of the mean-squared fluctuations and the mechanical stiffness should be examined. In addition to these, for multi-domain proteins, the *z*-components of the principal components should be examined in order to identify those modes that move the domains in opposite directions along the direction of the applied force. These modes are likely to be amplified by the application of the force in the given direction. The *z*-components of the principal components should be examined. To aid in selecting these modes, it may be helpful to calculate the mean of the *z*-components of the individual domains.
5. Examine the characteristic motions of each selected mode. Create pseudo-trajectories to view the effects of these modes on the protein’s overall motion. Often, only a subset of the selected modes will actually produce motions that are relevant for studying the effect of the applied forces. For example, in several cases, the *z*-components may show clear shifts in opposite directions, but these may arise from rotational motions rather than lateral shifts. Those modes that shift the positions of the domains primarily through lateral shifts should be selected and used to create distorted structures. How these distorted structures are generated will depend on the software package being used. For example, ProDy [33,34] calculates an ensemble of structures using a target RMSD, but with the direction and amplitudes of the individual modes chosen randomly. In this case, the resulting ensembles have to be evaluated statistically. ElNémo [35], on the other hand, allows individual conformations to be calculated by specifying the amplitude and direction of individual modes; in this case, it is sufficient to calculate only one or a few distorted structures. These distorted structures can then be examined to determine which domain or other part of the structure is likely to come apart first. This can be approximately quantized by following the volume of the buried residues of each distorted domain: the domain with the lowest overall volume of the buried core is likely to have the lowest structural integrity.

## 3. Results

### 3.1. T4 Lysozyme

**Figure 2:**
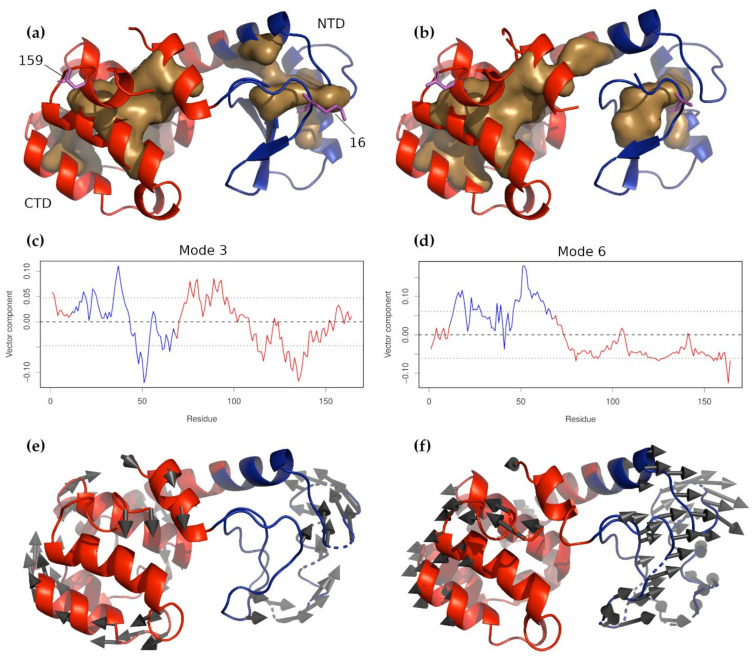
T4 Lysozyme and circularly permuted mutant CP13. (**a**) Wild-type T4 lysozyme and (**b**) CP13. In both panels, the N-terminal domain is colored blue, and the C-terminal domain is red. The buried cores of each domain are indicated by the light brown surfaces. Residues 16 and 159, where the molecule was tethered for the force experiments, are shown in magenta sticks. The *z*-components of non-zero normal modes 3 and 6 for wild-type T4 are shown in panels (**c**,**d**), respectively, with the residues corresponding to the NTD and CTD colored as in panels (**a**,**b**). The dashed line near 0.00 indicates the mean displacement. It can be seen, especially for mode 6, that the two domains moved in opposite directions in these modes, meaning that they were likely to be amplified by the applied force. The characteristic motions of these two modes are shown in panels (**e**,**f**) below their respective *z*-component plots (mode 3 in (**e**) and mode 6 in (**f**)).

The T4 phage lysozyme has been the subject of a truly staggering number of structure, function, stability, and folding studies [36]. The protein comprises two domains, a smaller one occupying the N-terminal part of the molecule (N-terminal domain, NTD), and a larger one on the C-terminal side (C-terminal domain, CTD). One structural feature is that residue 12 of the N-terminal forms an α-helix that is structurally part of the C-terminal domain (Figure 2a,b). To study the consequences of this arrangement, a number of structurally permuted mutants were prepared, which move this α-helix from the N-terminus to the C-terminus while keeping the overall fold of the structure, and the protein’s enzymatic activity, intact. The effect of this change on the folding of T4 lysozyme was studied by applying molecular pulling to wild-type T4 lysozyme and the circularly permuted mutant CP13 [37,38,39]. The results showed that the domains of the wild-type protein folded in a cooperative way, while those of the CP13 mutant did not, and that unfolding of the wild-type protein, especially its N-terminal domain, was more difficult than the mutant. During the pulling experiment, the lysozyme was linked to the apparatus through connections at residues 16 and 159. The results of the experiment were interpreted with the help of equilibrium and steered molecular dynamics simulations using both all-atom and coarse-grained force fields [40]. In attempting to analyze the results of these experiments with NMA, it was not reasonable to expect to answer questions of folding cooperativity; however, the prospect of establishing whether or not the N-terminal domain became more or less stable as a consequence of the mutation was more realistic. The PDB structures 2LZM [41] and 2O4W [42] were used as the wild-type and CP13 structures, respectively.

Initial examination showed the two separate domains with a node between them, suggesting that they would move independently. The N-terminal domain was also clearly smaller than the C-terminal one. The square fluctuations calculated from the first 100 non-zero normal modes showed that in both forms, the N-terminal domain was more flexible than the C-terminal domain and that the flexibility of the N-terminal domain was slightly higher in the CP13 mutant than in the wild-type (mean Cα fluctuations of 0.189 Å^2^ for the mutant vs. 0.181 Å^2^ for the wild-type). The relative stiffness of the N-terminal and C-terminal domains remained roughly the same in both forms, however.

After aligning the direction of applied force with the coordinate system *z*-axis and calculating the normal modes, the contributions of the *z* components of each of the first 50 normal modes were plotted (Figure 2c,d). Taking into consideration the collectivities of the individual modes, the results showed that the first nine non-zero modes were worth examining more carefully; therefore, pseudo-trajectories were created for each mode, which showed the molecular motions captured by each mode. (Figure 2e,f). The modes that most clearly moved the domains in the direction of the applied force were 3, 6, 7, and 8. These modes were used to construct a number of distorted structures. Because we wanted to determine the structural stabilities of the individual domains, the structures were distorted with an RMSD of 12 Å. The process by which these structures are created is stochastic: any given generated conformation may apply motions from one mode to move the domains apart and motions from a second to push them together. As a consequence, a suitably large number of conformations must be generated in order to adequately gauge the integrity of the individual domains. To follow the trends, we also progressively distorted the structures by producing smaller numbers of conformations at RMSD steps of 2, 4, 6, 8, 10, and 12 Å. Both approaches showed that the NTD unfolded more rapidly than the CTD and that this happened to a greater extent in the CP13 mutant. This could be partially quantified by calculating the volume of the residues buried by each domain for each RMSD step: the average buried volume could be taken to reflect how well packed the given domain was. Overall, the wild-type NTD buried 77 ± 160 Å^3^ while the CP13 buried 24.1 ± 70 Å^3^; when considering only those conformations where the buried volume was not zero, the wild-type NTD buried 255 ± 190 Å^3^ while CP13 buried only 97 ± 114 Å^3^. The RMSD ramp produced similar results, showing that for nearly all RMSDs considered, the buried volume of the wild-type was greater than that of the CP13 mutant. We can therefore conclude, in agreement with the experimental and computational studies, that the circular permutation does weaken the structural integrity of the NTD of T4 lysozyme.

### 3.2. Hsp70

The 70-kDa heat shock proteins (Hsp70) are ATP-dependent molecular chaperones that are found throughout all kingdoms of life, from archaebacteria and prokaryotes to eukaryotes and mammals. It is one of the most conserved proteins, with 40–60% identity between prokaryotic and eukaryotic homologues [43], and it plays key roles in protein folding, disaggregation, and degradation [44,45]. The *E. coli* homologue of the Hsp70 chaperone, DnaK, is probably the most thoroughly studied [46], and it has been extensively studied using optical trap experiments in our laboratory for a number of years [47,48,49,50]. Hsp70 can be divided into two major subunits, the nucleotide-binding domain (NBD) and the substrate-binding domain (SBD), both of which undergo conformational changes as a result of binding either the nucleotide or a substrate peptide.

#### 3.2.1. Nucleotide-Binding Domain

We studied the effect of nucleotide binding on the structural stability of the isolated NBD using single-molecule force spectroscopy, which was interpreted with the help of steered molecular dynamics simulation [47]. We found that nucleotide binding did not change the force needed to unfold the protein, but did change the pathway by which unfolding occurred. The Hsp70 NBD could be divided into two principal domains, each of which was composed of two subdomains or lobes. In the absence of nucleotide, the C-terminal α-helices were first extracted from the structure, followed by the unfolding of the C-terminal domain, called lobe II, and, last, the N-terminal domain, called lobe I. When bound to ATP, the first step was still the extraction of the C-terminal α-helix, but, in this case, lobe I unfolded before lobe II.

**Figure 3:**
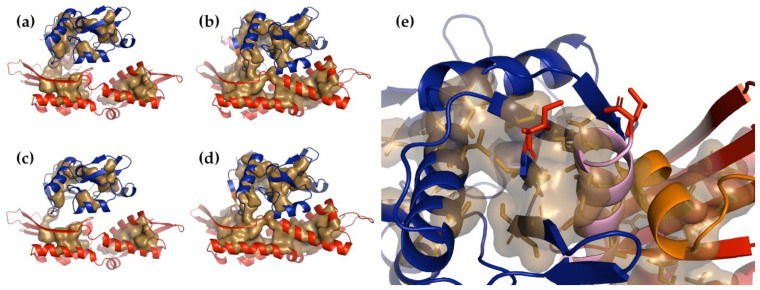
The Apo and Holo forms of the Hsp70 NBD. In all panels, lobe I is blue, lobe II is red, the C-terminal α-helix is pink, and the linker between the two domains is yellow. The sand-colored surface inside each molecule shows the residues buried in the domain core. (**a**) The intact Apo form, (**b**) the intact Holo form, (**c**) the tailless Apo form, and (**d**) the tailless Holo form. Note that loss of the C-terminal α-helix broke the density between the two domains, and NMA showed that lobe I and lobe II became more flexible with respect to one another. (**e**) The region of the NBD Apo form containing both the N- and C-terminal residues (as sticks, colored red). A careful examination suggests that applying force in opposite directions to the red-colored residues will result in the extraction of the C-terminal α-helix (pink). The Apo form is from PDB structure 2KHO [51] and the Holo form is from PDB structure 4B9Q [52].

Making use of our NMA protocol, we could essentially reproduce this analysis. By examining the distribution of buried residues in the Apo and Holo forms (Figure 3a–d), we could easily see the two primary domains and subdomains. In our experiments, the force was applied to the N- and C-terminal residues, which were spatially adjacent in the tertiary structure. Examining this region (Figure 3e) suggests that when the tension is applied in opposite directions to these two residues, the C-terminal α-helix is more likely to be extracted before the N-terminal β-strand. The N-terminus was at the end of the middle strand of a mixed β-sheet and was sandwiched between two α-helices, one of them the C-terminal α-helix. The C-terminal α-helix, on the other hand, was held in only by packing forces, not hydrogen bonds, and since the majority of the residues in this helix were glycines and alanines, those forces were not likely to be as strong as might otherwise be expected.

The distribution of buried residues in these two structures suggests that the structurally weakest points in the protein are likely to be the junctions between the subdomains and between lobes I and II. It is curious to note that the nucleotide-bound form appeared to have substantially more buried volume than the Apo form. While it is tempting to attribute all of this to the binding of the nucleotide, it is also possible that it arose because the two structures were determined using two different techniques: the Apo structure, which is less dense, was determined by NMR, while the denser Holo form was determined by X-ray crystallography. It seems quite possible that packing the protein into a lattice could have compressed it more than might have been the case in the NMR solution form. This does complicate the possibility of making comparisons between the two structures, but by assuming that the number of buried lobe I residues remains approximately constant, the binding of the nucleotide can be expected to increase the relative buried volume of lobe II by about 23%.

NMA was conducted on both the intact structure and on a “tailless” form lacking the C-terminal 23 residues. For the intact structure, many modes were found, which rotated various combinations of the subdomains with respect to one another, but in each of these modes, the C-terminal α-helix moved together with the N-terminal lobe of the NTD (structurally and dynamically, the C-terminal α-helix should probably be regarded as belonging to lobe I rather than lobe II). Similarly, the α-helix that forms the linker between lobe I and lobe II has dynamics that match those of the proximal lobe of the CTD. Only one of the top 50 modes with the highest collectivity moved the N-terminal and C-terminal residues in opposite directions (the 22nd non-zero mode), and it was found that deforming the structure progressively with increasing RMSD targets failed to separate the domains.

**Figure 4:**
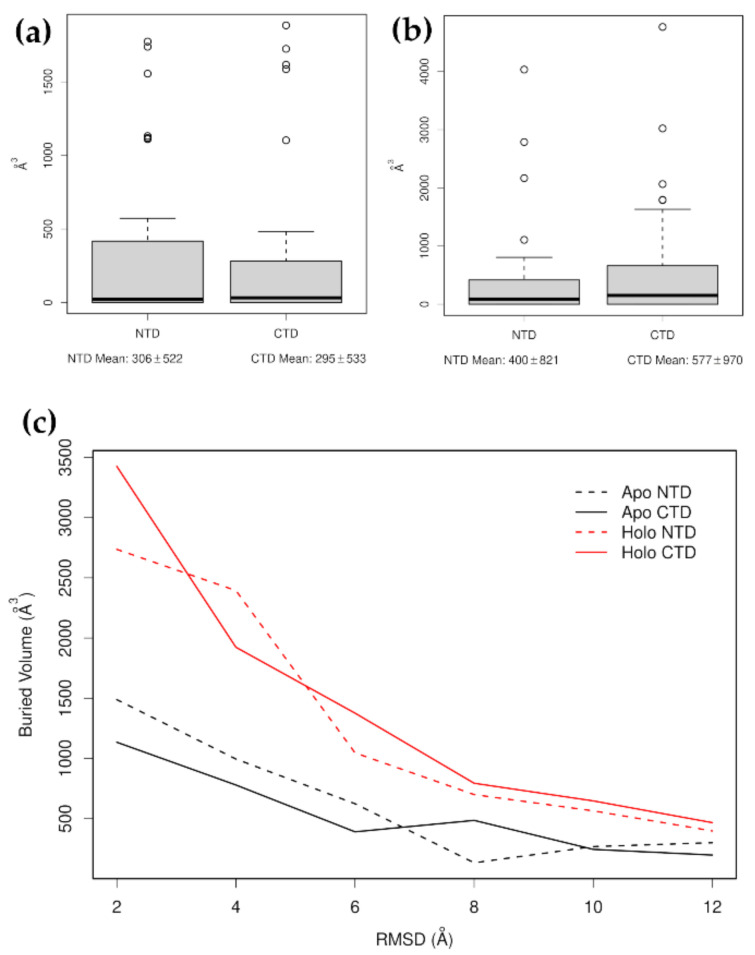
Plots of the buried volumes of lobe I and lobe II produced by structures distorted with a target RMSD of 12 Å, (**a**,**b**), and progressively with targets of 2, 4, 6, 8, 10, and 12 Å (**c**). It can be seen that in the Apo form, lobe I maintained a greater buried volume than lobe II. while the reverse was the case in the Holo form.

NMA on the tailless form identifies seven modes that separate lobe I and lobe II along the *z*-axis (non-zero modes 2, 4, 6, 8, 9, 10, and 23). Using these modes to deform the structures shows that lobe I maintained a higher buried volume than lobe II in the Apo form, while lobe II had the higher volume in the Holo form (Figure 4). Assuming that buried surface volume is correlated with structural integrity, this shows that in the Apo form, lobe II comes apart sooner than lobe I, while this situation is reversed in the Holo form. These are similar to, but less detailed and less specific than, the conclusions reached in our study using steered molecular dynamics simulation [47].

#### 3.2.2. Substrate-Binding Domain

The structural stability and unfolding pathways of the substrate-binding domain were also studied by single-molecule force spectroscopy [48]. The SBD has two domains, an N-terminal β-sheet domain (β-domain, residues 393–507) and a C-terminal α-helical domain (α-domain, residues 508–603). Our studies found that unfolding can proceed along one of two pathways: in approximately 1/3 of cases, the β-domain unfolded before the α-domain, while in 2/3 of cases, the α-domain unfolded first. In both pathways, one of the first steps was the release of the two C-terminal β-strands of the β-domain (strands β7 and β8).

**Figure 5:**
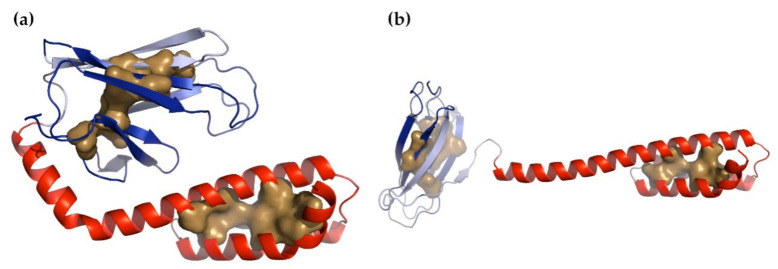
The compact and extended forms of the Hsp70 substrate binding domain. The β-domain is blue, the α-domain is red, and the two C-terminal β-strands of the β-domain (β7 and β8) are shown in light blue. (**a**) Compact, substrate-bound form (from PDB structure 2KHO [51]) and (**b**) extended form (from PDB structure 4B9Q [52]).

**Figure 6:**
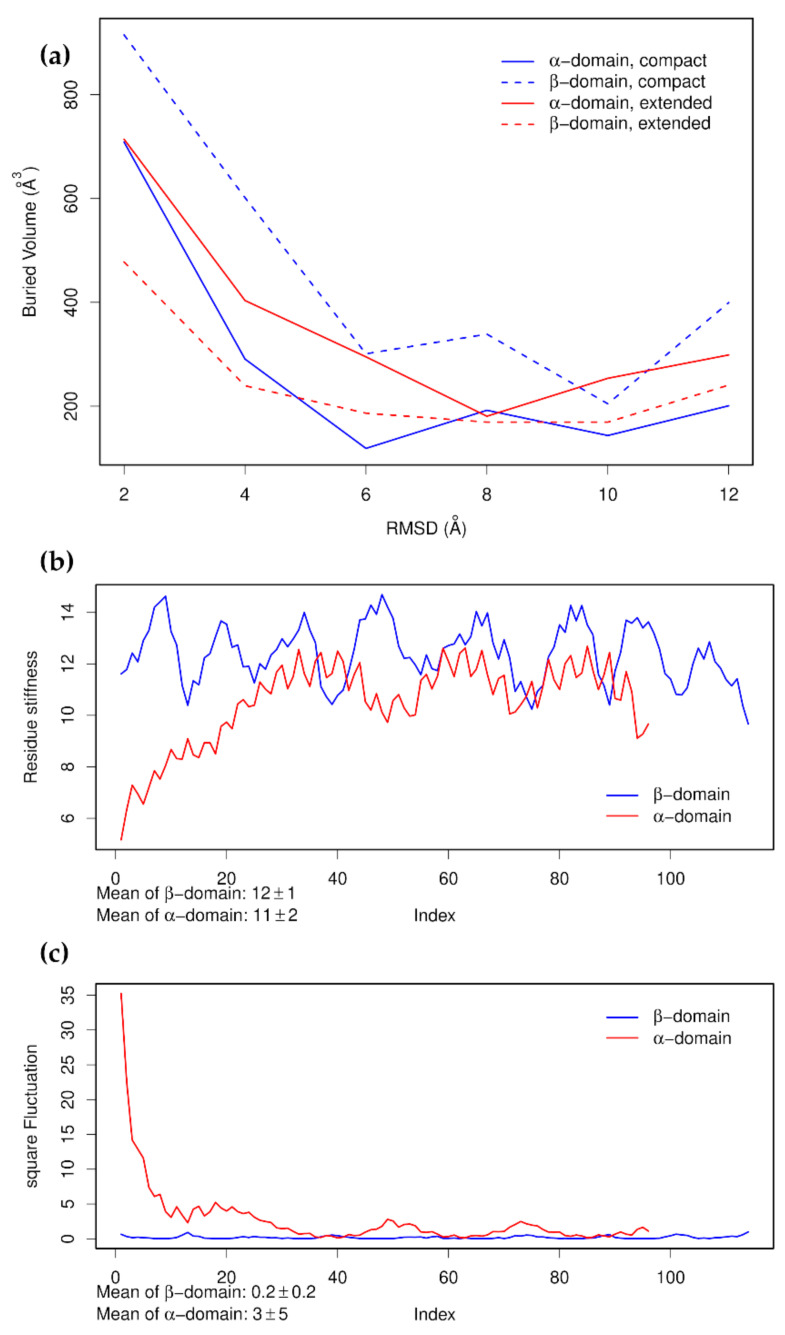
Plots showing (**a**) the buried volumes of the β-domain and α-domain produced by structures distorted with progressively increasing target RMSDs, (**b**) the stiffness of the α-domain and β-domain of the extended form of the SBD calculated separately, and (**c**) the square fluctuations produced by the first 100 non-zero normal modes of these two domains calculated separately. In all panels, the coloring of the domains matches that of Figure 5. It can be seen that although the buried volumes gave different answers for the two different starting structures, when the domains were examined separately, the α-domain was the more flexible one.

Two structural conformations are known for the Hsp70 SBD, a compact, substrate-bound form and an extended, unbound form (Figure 5). When binding a substrate, the long N-terminal α-helix of the α-domain broke and the α-domain wrapped around the β-domain. The β-domain underwent conformational changes, which greatly increased its buried volume. There was no buried volume between the α-domain and β-domain in the compact form, which suggests that if force were to be applied to the N- and C-terminal residues of the compact form, then the first step would be to unwrap the α-domain, converting the compact form to the extended form. Moreover, in the extended form, it can be seen that strand β8 became shortened and less well ordered. This also had the effect of increasing the exposure of β7. Carrying out NMA on the extended form produced many normal modes that reflected large, relatively local motions involving the long α-helix that connected the α- and β-domains. Non-zero modes 2, 3, 6, and 8 did show movements of the α- and β-domains away from one another along the *z*-axis. Distortion of the structure along these modes suggested that the β-domain was more likely to come apart before the α-domain. We reached the same conclusion when conducting the same analysis on the α- and β-domains separately. By carrying out this analysis on the compact form, we arrived at the opposite conclusion, that the α-domain is likely to come apart before the β-domain (Figure 6a). However, after calculating the normal modes for the α- and β-domains separately and examining the square fluctuations and the stiffness of the individual residues, we found that for both the compact and extended forms, the α-domain was always more flexible than the β-domain (Figure 6b,c). All these results are consistent with the results of our earlier study [48]; both domains could be the first to unfold and it was not, in principle, possible to predict in advance which of the two unfolding pathways was likely to be followed, though it was known that the α-domain did more often unfold first.

This highlights two limitations of this method under a specific set of conditions. First, the most stable domain, measured by buried volume, will often be found to be whichever starting structure also had the greatest buried volume. It is dependent upon the starting structure, and thus, all available starting structures, or at least a representative number of them, should be used. The second is that when multiple unfolding pathways are available for the same structure in the same state, only one is likely to be found.

### 3.3. Glucocorticoid Receptor

The glucocorticoid receptor (GCR) is a steroid hormone receptor, a soluble, ligand-controlled transcription factor that moves between the cytosol and the nucleus [53]. Similarly to the other members of this class, it has an N-terminal domain, a DNA-binding domain, and a ligand-binding domain [54]. A number of structures are available for the latter two domains; these are complexed to a variety of substrates [55,56], but not the N-terminal domain. The ligand-binding domain (LBD) of GCR is a single-domain, α-helical protein. Its folding pathway and hormone-binding properties were studied using single-molecule optical trap experiments [57]. The authors identified an N-terminal “lid” structure whose opening and closing were tightly coupled to substrate binding, but apparently without making direct contact with the hormone. They were also able to show that folding proceeded in the absence of hormone and that there were three unfolding intermediates.

Although there are a number of structures of the GCR-LBD available, they are all in the closed-hormone-bound form, owing largely to the fact that the hormone-free form is unstable and aggregation-prone [58] (indeed, it is strongly regulated by the chaperones Hsp70 and Hsp90 [59]). This means that NMA can proceed only from the closed and ligand-bound form and the full dynamics of the open form are unavailable. Given the possibly large change between the open, unbound form and the closed form, it may not be possible to recover the dynamics around ligand binding, although it may be possible to qualitatively reproduce the unfolding pathway.

**Figure 7:**
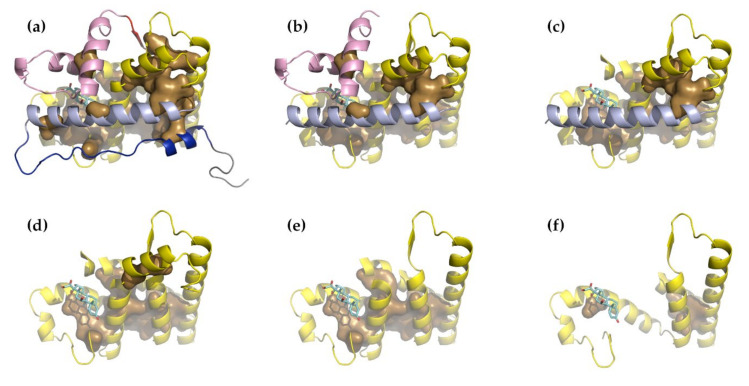
A series of truncations of the GCR-LBD suggested by considering the buried residue distribution and the results of NMA. (**a**) The whole GCR-LBD domain; (**b**) the first truncation, lacking NT1 and CT1; (**c**) the second truncation, lacking CT2; (**d**) the third truncation, lacking NT2; (**e**) a forth truncation, lacking residues 583–600 and 731–734; and (**f**) a fifth truncation, lacking residues 601–619 and 724–730. The structures are colored in the following way: blue: NT1, residues 530–554; light blue: NT2, 555–582; pink: CT2, 735–767; red: CT1, residues 768–777. The first 8 N-terminal residues (523–529, gray) were exceptionally mobile and dominated the first 10 non-zero normal modes and were removed from the analysis. The buried core residues are shown in sandy brown and the remaining core is yellow.

Examination of the distribution of buried residues (of PDB structure 1M2Z [55]) showed that a surprisingly large part of the molecule is at least partially solvent-exposed. In particular, two N-terminal regions (523–554, NT1 and 555–582, NT2) and two C-terminal regions (768–777, CT1 and 735–767, CT2) were found to bury relatively small volumes and are, therefore, likely to become unfolded relatively easily under force. Together, these comprise 101 of 255 residues (around 40% of the total) and expose about 52% of the buried volume (2433.72 Å^3^ remains of 5114.21 Å^3^). NMA was, therefore, carried out on a set of progressively truncated structures: the initial whole structure, a first truncation lacking NT1 and CT1, a second truncation missing CT2, and a third missing NT2. NMA of this last structure suggested an additional two truncations: the removal of residues 583–600 and 731–734, and the removal of residues 601–619 and 724–730 (Figure 7).

**Figure 8:**
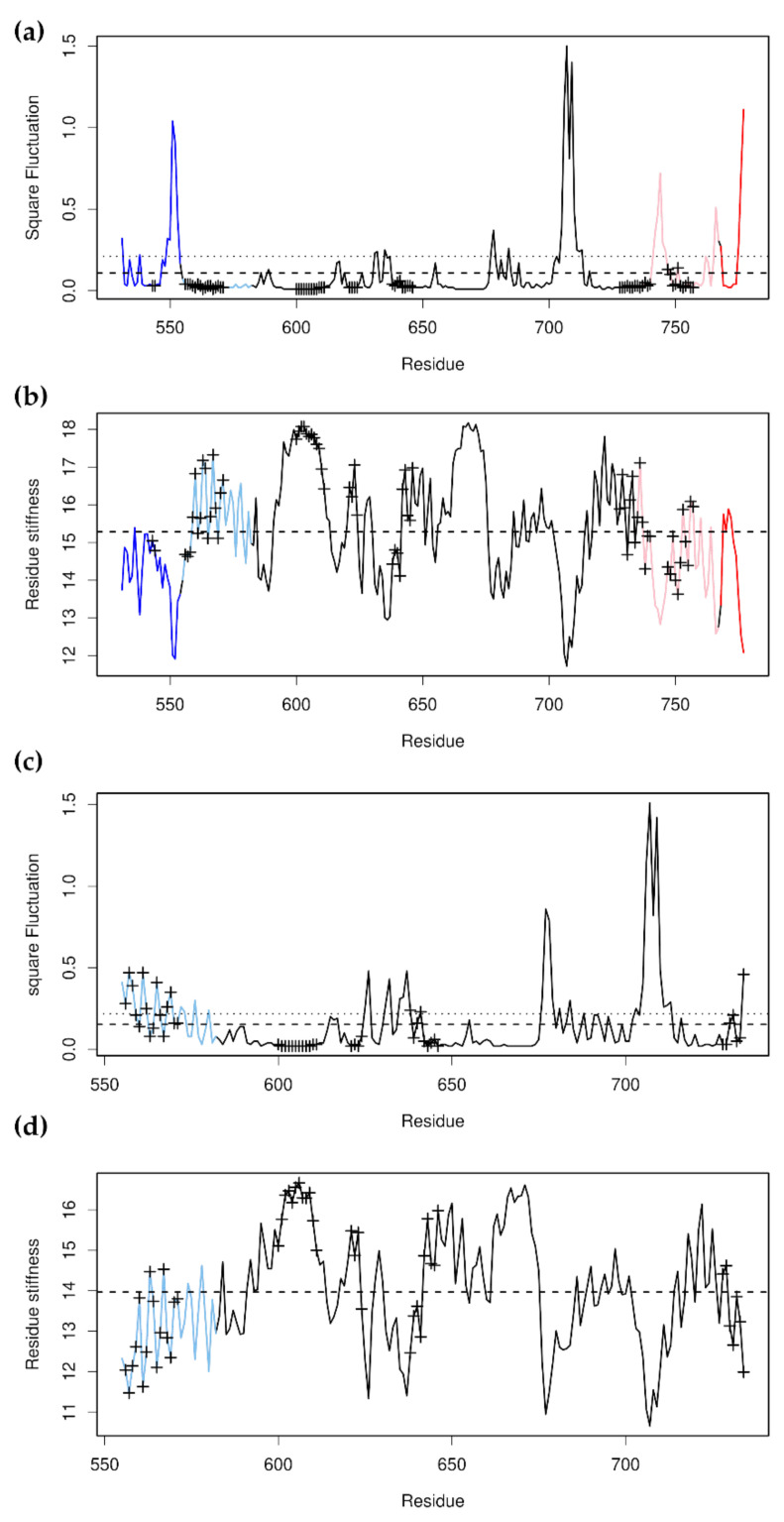
The overall square fluctuations and residue stiffness of the whole GCR-LBD (Figure 7a) and the second GCR-LBD truncation (Figure 7c). (**a**,**b**) show the square fluctuations and residue stiffness of the whole GCR-LBD, respectively, while (**c**,**d**) show the square fluctuations and residue stiffness, respectively, of the second GCR-LBD truncation. The plot is colored as in Figure 7. The crosses mark the residues that were within 8 Å of the ligand in PDB structure 1M2Z, and which were presumably part of the ligand-binding site. It can be seen that, discounting individual loops, NT1 and both CT1 and 2 had the greatest overall degree of flexibility and that the removal of NT1 and CT1 and CT2 greatly increased the flexibility of NT2. The consequences of this can be seen in Figure 9a below.

**Figure 9:**
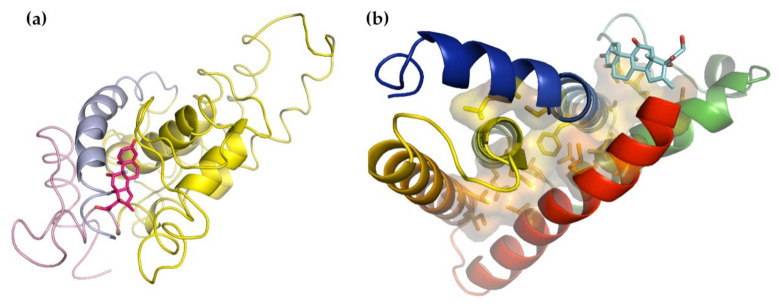
Fragments of the GCR-LBD. (**a**) A distortion of the first truncation (Figure 7b) following the 10 non-zero modes with the highest collectivity and an RMSD target of 2.0 Å. It can be seen that one effect of removing NT1 was the unraveling of the N-terminal part of NT2, which contained a large number of residues that were involved in binding the hormone. (**b**) The three-way link between α-helices 8, 7, and 5, forming the last major sticking point between two different unfolding intermediates.

NMA on the complete structure showed that the N-terminal 8 residues are, by far, the most flexible and effectively suppress the other motions of the first 8–10 non-zero normal modes (gray in Figure 7a); consequently, they were removed and the analysis reported for the whole structure will not have these. NMA on this structure showed that the most mobile part of the structure is the loop between α-helices 8 and 9 (residues 700–715; this region also had the highest *B*-factors in the crystal structure). The second-most flexible parts of the structure, however, were NT1 and CT1 and CT2. (CT2 contains the region corresponding to α-helix 12, which is known to be one of the most conformationally labile regions [56], so it is not surprising to find that it is the most movable part of the binding site.) This can be seen in plots of the square fluctuations and the residue stiffness (Figure 8a,b). NT2, on the other hand, is one of the more stable and less flexible parts of the structure. NT2 and CT2 both feature a large number of residues that are within 8 Å of the bound substrate in the crystal structure, and that presumably take part in substrate binding. Removing NT1 has the effect of increasing the flexibility of NT2, and removing CT2 increases it even more. The practical consequence of this is that removal of NT1 allows the N-terminal end of NT2 to become disordered and partially unraveled (Figure 8c,d and Figure 9a), thereby disrupting the substrate-binding site. This would account for the observed importance of the “lid” for substrate binding. Structurally, the “lid” provides a mechanical force that prevents the N-terminal residues of NT2 from unwinding, thereby holding the binding-site residues in place (indeed, it might be better to refer to it as a “lever” rather than a “lid”). This might also account for the observed importance of the LXXLL motif in residues 532–536 for ligand binding [60]: these residues form the N-terminal half of α-helix 1 and are important for anchoring NT1 to the rest of the protein (Figure 7a).

While truncations 1–3 could be arrived at by considering only the distribution of buried residues, further truncation, representing further unfolding, required NMA. NMA on the remaining portion (Figure 7e and Figure 9b) showed that the three-way connection between α-helices 8, 7, and 5 ties this part of the structure together and that removing it is likely to be the last sticking point separating one folding intermediate from another. Although many of the buried residues in this area were branched or aromatic, once the first break was made, unburying Phe-606 in α-helix 5, there was not a high likelihood of significant resistance since many of the remaining ones were quite small, or could be easily separated given the direction of the applied force.

Finally, Suren et al. [57] were able to detect three unfolding intermediates together with the opened and closed forms of the structure. These held 245 ± 9, 212 ± 8, 172 ± 11, 145 ± 10, and 101 ± 12 folded residues for the closed, open, intermediate 1, intermediate 2, and intermediate 3 forms, respectively. These correspond quite closely to our whole (246 residues, Figure 7a), truncation 1 (213 residues, Figure 7b), truncation 2 (180 residues, Figure 7c), truncation 3 (152 residues, Figure 7d) or truncation 4 (130 residues, Figure 7e), and truncation 5 (104 residues, Figure 7f) structures, respectively. These truncated forms were created without prior knowledge of the results of this study, and thus, might be tentatively taken to represent the residues involved for each intermediate form.

## 4. Discussion

Single-molecule force spectroscopy of proteins provides detailed insights into the mechanisms of protein folding/unfolding. The primary outcomes of single-molecule experiments are force distributions and distance changes. While forces and distances can be used for assessing the energetics of the folding/unfolding processes, distances can be additionally transformed into contour-length changes using worm-like chain model analysis. Researchers use contour lengths to estimate the number of amino acids involved in a given folding/unfolding transition. In subsequent steps in the research, the production of protein variants either confirms or refutes the structure-based hypothesis of the folding/unfolding steps. Structural interpretation and hypothesis formulation often require reasonable guesses. These are based on an inspection of the three-dimensional structure or, if available, from the results obtained by steered molecular dynamics simulation [47,48,49,50,61,62]—a computationally highly demanding technique that requires specialized expertise and training. Clearly, a simpler tool for gaining insights into the structural mechanics of proteins would benefit researchers in the single-molecule force spectroscopy field. In particular, the identification of mechanically vulnerable sites and stable/unstable interfaces can provide a versatile basis for producing the protein variants needed for detailed insights into the microscopic steps of the folding/unfolding mechanism.

In this study, we developed a procedure for using normal mode analysis to aid in interpreting the results of single-molecule force spectroscopy experiments. We revisited four previously published single-molecule force microscopy experiment studies, applied our procedure to each of them, and managed to recover many of the major findings, albeit in a qualitative way. Using three non-homologous proteins—T4L, Hsp70 and GCR—we found that, overall, NMA can function as a partial substitute for steered molecular dynamics simulation and can usefully supplement interpretations that arise from worm-like chain models.

T4 lysozyme is a model protein for folding studies [36,63]. It is composed of two domains that are discontinuous in sequence, which may already imply some degree of structural and energetic coupling between domains (see, for example, bacterial SlyD protein, with similar discontinuous topology [64]). In traditional bulk chemical denaturation, such energetic coupling and cooperativity are difficult to assess due to global changes in domain stability. To this end, wild-type, as well as a circularly permutated variant, CP13, which possesses otherwise similar structure, activity and global stability [42,65], was examined by single-molecule force spectroscopy. Circular permutation produces significant changes in the order of structural elements along the sequence and affects the coupling and folding cooperativity between its domains. Compared to the wild-type variant, when force is applied to the same residues in this variant, only the selected region is directly under load. By applying Crooks’ fluctuation theorem, Shank et al. [39] determined the free energy changes during mechanical unfolding, and they found complex folding trajectories for the CP13 variant with a lower degree of coupling between domains. The decoupling of the N-terminal domain from the rest of T4L results in significant destabilization that is in line with our NMA results and comparative buried area analysis of T4L and CP13.

Heat shock protein 70, Hsp70, consists of two domains—a nucleotide-binding domain and a substrate-binding domain. The mechanical properties of these Hsp70 domains under tension were investigated in two separate studies [47,48]. For the NBD, we found that mechanical unfolding is a cooperative process that involves first extracting the C-terminal gluing helix, thereby producing transiently populated intermediates with a cracked interface between lobe I and lobe II, and subsequent unfolding of the lobes. The subsequent unfolding of intermediates depends on the presence of nucleotides; in the Apo form, lobe II unfolds first, while in the Holo form, it is lobe I that is less stable. This exchange in the mechanical hierarchy of the domains is due to the unequal contribution of the nucleotide to the stability of the individual lobes. The presence of a binding pocket in lobe II accommodates the binding of the adenosine and ribose moieties, while lobe I senses the phosphorylation status (triphosphate/diphosphate) of the nucleotide. The exchange in the mechanical hierarchy can also be inferred from the results of the NMA. In particular, plots of the buried volumes of lobe I and lobe II produced by the distorted structures in Figure 4 show the switching between the physical properties of lobes. For the SBD, we found, in our previous study [48], that unfolding of the SBD can be described by bifurcated pathways consisting of the unfolding of the α-helical and β-subdomains. Before the unfolding of either domain, we observed structural fluctuations at 10 pN due to the rapid opening and closing of the domain interface. Opening and closing of the domain interface is coupled to peptide substrate binding and can be described as a Brownian ratchet motion. In this paper, we found, using NMA, that the non-zero modes 2, 3, 6, and 8 do show movements of the α- and β-subdomains away from one another. Again, the NMA results recover the experimental results on SBD nanomechanics. Concerning the mechanical unfolding of the SBD subdomains, either domain could be the first to unfold, with a slight preference for the initial unfolding of the α-subdomain. It should be noted, however, that both unfolding pathways could be identified by this method only because two different conformations of the SBD were available. If only one of them had been available, then this method would have only been able to detect one of them. Generally, when there is a bifurcated unfolding pathway, this method is unlikely to be able to detect more than one branch unless there are initial structures available that favor both branches.

The glucocorticoid receptor, GCR, is a member of the nuclear receptor superfamily. GCR consists of three domains, the N-terminal activation function-1 domain, a DNA-binding domain (DBD), and a ligand-binding domain (LBD). Single-molecule force spectroscopy experiments were conducted on the LBD along the N-to-C terminal pulling directions. The LBD all-α structure consists of a canonical three-layered α-helical fold that entirely surrounds a ligand in the ligand-binding cavity. The binding/unbinding kinetics of the ligand are accompanied by the motion of the lid structure, whose opening and closing are tightly coupled to hormone binding. Surprisingly, this lid is located at the N-terminus without direct contact with the hormone [57]. From all the proteins described above, T4L and Hsp70, folding of the LBD is the most complex and involves at least three intermediates (IM1-3), and complete refolding in the absence of force is inefficient due to the formation of misfolded structures. Based on the workflow employing NMA and truncation, which was successfully applied for T4L and Hsp70, we identified potential structural candidates for intermediates of GCR LBD. These potential mechanical intermediates can be used for additional hypothesis-driven investigation into the folding and unfolding pathways of the GCR LBD.

As can be seen from these four studies, the topology of the given protein and the direction of applied force in the experiment greatly influence the nature of the analysis. The GCR LBD is a single-domain protein, while T4 lysozyme and Hsp70 are multidomain proteins. For T4 lysozyme and the two Hsp70 subdomains, it was sufficient to calculate distorted structures using the normal modes that were aligned in the direction of the applied force, but for the GCR LBD, it was necessary to sequentially peel away the outer layers of the protein. As another example, there exists another force-spectroscopy study on the Hsp70-NBD [50], in which the force is applied to lobe Ib and lobe IIb rather than to the N- and C-termini. In this experimental arrangement, the C-terminal α-helix would not have been extracted as the first step of the analysis, and a different series of normal modes would have been calculated from a different structure.

Our method does have some similarities to one form of the structural perturbation method developed by Zheng, Brooks, and Thirumalai [66,67,68,69]. The general idea behind this method is that the dynamic importance of a given position in a particular mode can be ascertained by calculating its response to a given perturbation. Using this technique, they explored the structural transitions in DNA polymerase [66,67], GroEL [67,68], and Myosin II [67,69]. They discovered that a number of conserved residues that were not otherwise close to the catalytic sites of these proteins nevertheless had important roles in their dynamics. Analogously, we found that a number of the most stable residues in the three proteins studied here had the greatest stiffness and lowest square fluctuations. They also found that the modes which appeared to be most important for functional dynamics were robust to changes in the protein amino-acid sequence that did not disrupt the network of connections between the most important dynamic residues. Some functionally important modes were found not to be robust by themselves but did form part of a robust group of modes that did not mix well with other modes upon perturbation, and they speculated that these groups of modes might be used to provide variations on a general functional dynamic motion across different isoforms of the same protein [69]. Analogously, in the method described here, we suggest that a group of normal modes whose dynamic motion corresponds to the direction of the applied force is amplified by the applied force.

## 5. Conclusions

We conclude that applying NMA and buried volume analysis to the native structure of proteins can provide a rational basis for predicting the mechanical states of proteins observed in single-molecule force spectroscopy. Generally, native proteins have a small distance to the transition state, <2 nm, with small deformations resulting in unfolding, so-called brittle, behavior [70]. The apparently good agreement between the mechanical experiments and NMA might arise from the mechanically brittle behavior of protein structures, thereby allowing their large amplitude motions to be well described by a harmonic approximation such as that produced by NMA.

## Data Availability

Not applicable.

## References

[1] Kellermayer M.S., Smith S.B., Granzier H.L., Bustamante C. (1997). Folding-unfolding transitions in single titin molecules characterized with laser tweezers. Science.

[2] Rief M., Gautel M., Oesterhelt F., Fernandez J.M., Gaub H.E. (1997). Reversible unfolding of individual titin immunoglobulin domains by AFM. Science.

[3] Schuler B., Lipmanm E.A., Eaton W.A. (2002). Probing the free-energy surface for protein folding with single-molecule fluorescence spectroscopy. Nature.

[4] Rhoades E., Gussakovsky E., Haran G. (2003). Watching proteins fold one molecule at a time. Proc. Natl. Acad. Sci. USA.

[5] Neupane K., Foster D.A., Dee D.R., Yu H., Wang F., Woodside M.T. (2016). Direct observation of transition paths during the folding of proteins and nucleic acids. Science.

[6] Greenleaf W.J., Woodside M.T., Block S.M. (2007). High-resolution, single-molecule measurements of biomolecular motion. Annu. Rev. Biophys. Biomol. Struct..

[7] Mashaghi A., Bezrukavnikov S., Minde D.P., Wentink A.S., Kityk R., Zachmann-Brand B., Mayer M.P., Kramer G., Bukau B., Tans S.J. (2016). Alternative modes of client binding enable functional plasticity of Hsp70. Nature.

[8] Del Rio A., Perez-Jimenez R., Liu R., Roca-Cusachs P., Fernandez J.M., Sheetz M.P. (2009). Stretching single talin rod molecules activates vinculin binding. Science..

[9] Jagannathan B., Marqusee S. (2013). Protein Folding and Unfolding Under Force. Biopolymers.

[10] Crooks G.E. (1999). Entropy production fluctuation theorem and the nonequilibrium work relationfor free energy differences. Phys. Rev. E Stat. Phys. Plasmas Fluids Relat. Interdiscip. Topics.

[11] Collin D., Ritort F., Jarzynski C., Smith S.B., Tinco I., Bustamante C. (2005). Verification of the Crooks fluctuation theorem and recovery of RNA folding free energies. Nature.

[12] Dudko O.K., Hummer G., Szabo A. (2006). Intrinsic Rates and Activation Free Energies from Single-Molecule Pulling Experiments. Phys. Rev. Lett..

[13] Dudko O.K., Hummer G., Szabo A. (2008). Theory, analysis, and interpretation of single-moleculeforce spectroscopy experiments. Proc. Natl. Acad. Sci. USA.

[14] Puchner E.M., Franzen G., Gautel M., Gaub H.E. (2008). Comparing Proteins by Their Unfolding Pattern. Biophys. J..

[15] Hyeon C., Dima R.I., Thirumalai D. (2006). Pathways and Kinetic Barriers in MechanicalUnfolding and Refolding of RNA and Proteins. Structure.

[16] Zhmurov A., Dima R.I., Kholodov Y., Barsegov V. (2010). SOP-GPU: Accelerating biomolecular simulations in the centisecond timescale using graphics processors. Proteins.

[17] Lee E.H., Hsin J., Sotomayor M., Comellas G., Schulten K. (2009). Discovery Through the Computational Microscope. Structure.

[18] Ackbarow T., Chen X., Keten S., Buehler M.J. (2007). Hierarchies, multiple energy barriers, and robustness govern the fracture mechanics of α-helical and β-sheet protein domains. Proc. Natl. Acad. Sci. USA.

[19] Lichter S., Rafferty B., Flohr Z., Martini A. (2012). Protein High-Force Pulling Simulations Yield Low-Force Results. PLoS ONE.

[20] Kouza M., Hu C.K., Li M.S., Kolinski A. (2013). A structure-based model fails to probe the mechanical unfolding pathways of the titin i27 domain. J. Chem. Phys..

[21] Rico F., Gonzalez L., Casuso I., Puig-Vidal M., Scheuring S. (2013). High-Speed Force Spectroscopy Unfolds Titin at the Velocity of Molecular Dynamics Simulations. Science.

[22] Kmiecik S., Wabik J., Kolinski M., Kouza M., Kolinski A., Liwo A. (2014). Coarse-Grained Modeling of Protein Dynamics. Computational Methods to Study the Structure and Dynamics of Biomolecules and Biomolecular Processes.

[23] Bauer J.A., Pavlović J., Bauerová-Hlinková V. (2019). Normal Mode Analysis as a Routine Part of a Structural Investigation. Molecules.

[24] Skjaerven L., Martinez A., Reuter N. (2011). Principal component and normal mode analysis of proteins; a quantitative comparison using the GroEL subunit. Proteins.

[25] Rueda M., Chacón P., Orozco M. (2007). Thorough Validation of Protein Normal Mode Analysis: A Comparative Study with Essential Dynamics. Structure.

[26] Rueda M., Ferrer-Costa C., Meyer T., Pérez A., Camps J., Hospital A., Gelpi J.L., Orozco M. (2007). A consensus view of protein dynamics. Proc. Natl. Acad. Sci. USA.

[27] Doruker P., Atilgan A.R., Bahar I. (2000). Dynamics of Proteins Predicted by Molecular Dynamics Simulations and Analytical Approaches: Application to α-Amylase Inhibitor. Proteins.

[28] Knapp E.W., Fischer S.F., Parak F. (1982). Protein Dynamics from Mössbauer Spectra. The Temperature Dependence. J. Phys. Chem..

[29] Zaccai G. (2000). How Soft Is a Protein? A Protein Dynamics Force Constant Measured by Neutron Scattering. Science.

[30] Roh J.H., Novikov V.N., Gregory R.B., Curtis J.E., Chowdhuri Z., Sokolov A.P. (2005). Onsets of Anharmonicity in Protein Dynamics. Phys. Rev. Lett..

[31] Eyal E., Bahar I. (2008). Toward a Molecular Understanding of the Anisotropic Response of Proteins to External Forces: Insights from Elastic Network Models. Biophys. J..

[32] Mikulska-Ruminska K., Kulik A.J., Benadiba C., Bahar I., Dietler G., Nowak W. (2017). Nanomechanics of multidomain neuronal cell adhesion protein contactin revealed by single molecule AFM and SMD. Sci. Rep..

[33] Bakan A., Meireles L.M., Bahar I. (2011). *ProDy*: Protein Dynamics Inferred from Theory and Experiment. Bioinformatics.

[34] Bakan A., Dutta A., Mao W., Liu Y., Chennubhotla C., Lezon T.R., Bahar I. (2014). *Evol* and *ProDy* for bridging protein sequence evolution and structural dynamics. Bioinformatics.

[35] Suhre K., Sanejouand Y.H. (2004). *ElNémo*: A normal mode web server for protein movements analysis and the generation of templates for molecular replacement. Nucleic Acids Res..

[36] Baase W.A., Liu L., Tronrud D.E., Matthews B.W. (2010). Lessons from the lysozyme of phage T4. Protein Sci..

[37] Yang G., Cecconi C., Baase W.A., Vetter I.R., Breyer W.A., Haack J.A., Matthews B.W., Dahlquist F.W., Bustamante C. (2000). Solid-state synthesis and mechanical unfolding of polymers of T4 lysozyme. Proc. Natl. Acad. Sci. USA.

[38] Peng Q., Li H. (2008). Atomic force microscopy reveals parallel mechanicalunfolding pathways of T4 lysozyme: Evidence for a kinetic partitioning mechanism. Proc. Natl. Acad. Sci. USA.

[39] Shank E.A., Cecconi C., Dill J.W., Marqusee S., Bustamante C. (2010). The folding cooperativity of a protein is controlled by its chain topology. Nature.

[40] Zheng W., Glenn P. (2015). Probing the folded state and mechanical unfolding pathways of T4 lysozyme using all-atom and coarse-grained molecular simulation. J. Chem. Phys..

[41] Weaver L.H., Matthews B.W. (1987). Structure of bacteriophage T4 lysozyme refined at 1.7 Å resolution. J. Mol. Biol..

[42] Cellitti J., Llinas M., Echols N., Shank E.A., Gillespie B., Kwon E., Crowder S.M., Dahlquist F.W., Alber T., Marqusee S. (2007). Exploring subdomain cooperativity in T4 lysozyme I: Structural and energetic studies of a circular permutant and protein fragment. Protein Sci..

[43] Karlin S., Brocchieri L. (1998). Heat Shock Protein 70 Family: Multiple Sequence Comparisons, Function, and Evolution. J. Mol. Evol..

[44] Fernáandez-Fernáandez M.R., Gragera M., Ochoa-Ibarrola L., Quintana-Gallardo L., Valpuesta J.M. (2017). Hsp70—A master regulator in protein degradation. FEBS Lett..

[45] Havalová H., Ondrovičová G., Keresztesová B., Bauer J.A., Pevala V., Kutejová E., Kunová N. (2021). Mitochondrial HSP70 Chaperone System—The Influence of Post-translational Modifications and Involvement in Human Disease. Int. J. Mol. Sci..

[46] Genevaux P., Georgopoulos C., Kelley W.L. (2007). The Hsp70 chaperone machines of Escherichia coli: A paradigm for the repartition of chaperone functions. Mol. Microbiol..

[47] Bauer D., Merz D.R., Pelz B., Theisen K.E., Yacyshyn G., Mokranjac D., Dima R.I., Rief M., Žoldák G. (2015). Nucleotides regulate the mechanical hierarchy between subdomains of the nucleotide binding domain of the Hsp70 chaperone DnaK. Proc. Natl. Acad. Sci. USA.

[48] Mandal S.S., Merz D.R., Buchsteiner M., Dima R.I., Rief M., Žoldák G. (2017). Nanomechanics of the substrate binding domain of Hsp70 determine its allosteric ATP-induced conformational change. Proc. Natl. Acad. Sci. USA.

[49] Bauer D., Meinhold S., Jakob R.P., Stigler J., Merkel U., Maier T., Rief M., Žoldák G. (2018). A folding nucleus and minimal ATP binding domain of Hsp70 identified by single-molecule force spectroscopy. Proc. Natl. Acad. Sci. USA.

[50] Meinhold S., Bauer D., Huber J., Merkel U., Weißl A., Žoldák G., Rief M. (2019). An Active, Ligand-Responsive Pulling Geometry Reports on Internal Signaling between Subdomains of the DnaK Nucleotide-Binding Domain in Single-Molecule Mechanical Experiments. Biochemistry.

[51] Bertelsen E.B., Chang L., Gestwicki J.E., Zuiderweg E.R.P. (2009). Solution conformation of wild-type *E. coli* Hsp70 (DnaK) chaperone complexed with ADP and substrate. Proc. Natl. Acad. Sci. USA.

[52] Kityk R., Kopp J., Sinning I., Mayer M.P. (2012). Structure and Dynamics of the ATP-Bound Open Conformation of Hsp70 Chaperones. Mol. Cell.

[53] Weikum E.R., Knuesel M.T., Ortlund E.A., Yamamoto K.R. (2017). Glucocorticoid receptor control of transcription: Precision and plasticity via allostery. Nat. Rev. Mol. Cell Biol..

[54] Giguère V., Hollenberg S.M., Rosenfeld M.G., Evans R.M. (1986). Functional domains of the human glucocorticoid receptor. Cell.

[55] Bledsoe R.K., Montana V.G., Stanley T.B., Delves C.J., Apolito C.J., McKee D.D., Consler T.G., Parks D.J., Stewart E.L., Willson T.M. (2002). Crystal Structure of the Glucocorticoid Receptor Ligand Binding Domain Reveals a Novel Mode of Receptor Dimerization and Coactivator Recognition. Cell.

[56] Schoch G.A., D’Arcy B., Stihle M., Burger D., Bär D., Benz J., Thoma R., Ruf A. (2010). Molecular Switch in the Glucocorticoid Receptor: Active and Passive Antagonist Conformations. J. Mol. Biol..

[57] Suren T., Rutz D., Mößmer P.M., Merkel U., Buchner J., Rief M. (2018). Single-molecule force spectroscopy reveals folding steps associated with hormone binding and activation of the glucocorticoid receptor. Proc. Natl. Acad. Sci. USA.

[58] Lorenz O.R., Freiburger L., Rutz D.A., Krause M., Zierer B.K., Alvira S., Cuéllar J., Valpuesta J.M., Madl T., Sattler M. (2014). Modulation of the Hsp90 Chaperone Cycle by a Stringent Client Protein. Mol. Cell.

[59] Vandevyver S., Dejager L., Libert C. (2012). On the Trail of the Glucocorticoid Receptor: Into the Nucleus and Back. Traffic.

[60] Dong D.D., Jewell C.M., Bienstock R.J., Cidlowski J.A. (2006). Functional analysis of the LXXLL motifs of the human glucocorticoid receptor: Association with altered ligand affinity. J. Steroid Biochem. Mol. Biol..

[61] He C., Genchev G.Z., Lu H., Li H. (2012). Mechanically untying a protein slipknot: Multiple pathways revealed by force spectroscopy and steered molecular dynamics simulations. J. Am. Chem. Soc..

[62] Peng Q., Zhuang S., Wang M., Cao Y., Khor Y., Li H. (2009). Mechanical design of the third FnIII domain of tenascin-C. J. Mol. Biol..

[63] Matthews B.W. (1996). Structural and genetic analysis of the folding and function of T4 lysozyme. FASEB J..

[64] Žoldák G., Carstensen L., Scholz C., Schmid F.X. (2009). Consequences of domain insertion on the stability and folding mechanism of a protein. J. Mol. Biol..

[65] Llinás M., Marqusee S. (1998). Subdomain interactions as a determinant in the folding and stability of T4 lysozyme. Protein Sci..

[66] Zheng W., Brooks B.R., Doniach S., Thirumalai D. (2005). Network of Dynamically Important Residues in the Open/Closed Transition in Polymerases Is Strongly Conserved. Structure.

[67] Zheng W., Brooks B.R., Thirumalai D. (2006). Low-frequency normal modes that describe allosteric transitions in biological nanomachines are robust to sequence variations. Proc. Natl. Acad. Sci. USA.

[68] Zheng W., Brooks B.R., Thirumalai D. (2007). Allosteric Transitions in the Chaperonin GroEL are Captured by a Dominant Normal Mode that is Most Robust to Sequence Variations. Biophys. J..

[69] Zheng W., Thirumalai D. (2009). Coupling between Normal Modes Drives Protein Conformational Dynamics: Illustrations Using Allosteric Transitions in Myosin II. Biophys. J..

[70] Elms P.J., Chodera J.D., Bustamante C., Marqusee S. (2012). The molten globule state is unusually deformable under mechanical force. Proc. Natl. Acad. Sci. USA.

